# In Situ Fixation and Intertrochanteric Osteotomy for Severe Slipped Capital Femoral Epiphysis Following Femoral Neck Fracture: A Case Report with Application of Virtual Surgical Planning and 3D-Printed Patient-Specific Instruments

**DOI:** 10.3390/jpm15010013

**Published:** 2025-01-01

**Authors:** Giovanni Trisolino, Grazia Chiara Menozzi, Alessandro Depaoli, Olaf Stefan Schmidt, Marco Ramella, Marianna Viotto, Marco Todisco, Massimiliano Mosca, Gino Rocca

**Affiliations:** 1 Unit of Pediatric Orthopedics and Traumatology, IRCCS Istituto Ortopedico Rizzoli, 40136 Bologna, Italy; giovanni.trisolino@ior.it (G.T.); graziachiara.menozzi@ior.it (G.C.M.); marco.ramella@ior.it (M.R.); marianna.viotto@ior.it (M.V.); marco.todisco@ior.it (M.T.); gino.rocca@ior.it (G.R.); 2 Rizzoli Sicilia Department, IRCCS Istituto Ortopedico Rizzoli, 90011 Bagheria, Italy; 3 Department of Orthopedic and Traumatology, Franz Tappeiner Hospital—ASDAA Azienda Sanitaria Alto Adige, 39012 Merano, Italy; olafstefan.schmidt@sabes.it; 4 Orthopaedic Department, IRCCS Istituto Ortopedico Rizzoli, 40010 Bentivoglio, Italy; massimiliano.mosca@ior.it

**Keywords:** slipped capital femoral epiphysis, SCFE, femoral neck fracture, Imhäuser intertrochanteric osteotomy, 3D printing, virtual surgical planning, patient-specific instruments, CUX1

## Abstract

**Background**: Femoral neck fractures are rare but serious injuries in children and adolescents, often resulting from high-energy trauma and prone to complications like avascular necrosis (AVN) and nonunion. Even rarer is the development of slipped capital femoral epiphysis (SCFE) following femoral neck fracture, which presents unique diagnostic and treatment challenges. SCFE can destabilize the femoral head, with severe cases requiring complex surgical interventions. **Case presentation**: This report details a case of a 15-year-old male with autism spectrum disorder (ASD) who developed severe SCFE one month after treatment for a Delbet type III femoral neck fracture. The condition was managed with an Imhäuser intertrochanteric osteotomy (ITO), in situ fixation (ISF), and osteochondroplasty (OChP), supported by virtual surgical planning (VSP) and 3D-printed patient-specific instruments (PSIs) for precise correction and fixation. **Discussion**: The surgery was completed without complications. Six months after the operation, the patient exhibited a pain-free, mobile hip with radiographic evidence of fracture healing and no signs of AVN. Functional outcomes were favorable despite rehabilitation challenges due to ASD. **Conclusions**: The Imhäuser ITO, combined with ISF and OChP, effectively addressed severe SCFE after femoral neck fracture, minimizing AVN risk. VSP and PSIs enhanced surgical accuracy and efficiency, demonstrating their value in treating rare and complex pediatric orthopedic conditions.

## 1. Introduction

Femoral neck fractures in children and adolescents are rare but serious injuries, typically resulting from high-energy trauma and representing less than 1% of all pediatric fractures [1]. These fractures are classified using the Delbet system, which categorizes them based on fracture location: Type I (transepiphyseal), Type II (transcervical), Type III (cervicotrochanteric), and Type IV (intertrochanteric) [2]. Pediatric femoral neck fractures are prone to complications, including avascular necrosis, nonunion, and premature physeal closure, which can affect growth and hip function [2]. Avascular necrosis (AVN) remains the most serious complication of these fractures, in particular in Delbet Type II and Type III, with incidence rates ranging from 6% in nondisplaced fractures to 35% in displaced fractures, independent of the treatment approach [3].

Very rarely, femoral neck fractures may be complicated by slipped capital femoral epiphysis (SCFE) [1]. Spence et al. reported a 3% incidence of SCFE in a large case series of femoral neck fractures with an average of 2.8 years of follow-up [3]. Similarly to idiopathic SCFE, post-traumatic SCFE is characterized by a posterior and inferior displacement of the femoral head relative to the femoral neck, and it requires careful assessment of hip pain in affected patients using appropriate radiological studies [1].

SCFE classification is traditionally based on several criteria: stability (stable vs. unstable), degree of displacement (mild, moderate, or severe), and duration of symptoms (acute, chronic, or acute-on-chronic). The most relevant aspect is to differentiate a stable SCFE, which allows for weight-bearing without severe pain, from an unstable SCFE, which involves significant pain and instability, posing a higher risk for AVN [4]. For isolated SCFE, in situ fixation, without attempting to reduce the slippage, is the most widely accepted surgical treatment, particularly for mild to moderate chronic cases, which are the most common [5,6]. Open or closed reduction is rarely recommended for acute unstable slippage. Intra-articular and extra-articular osteotomies are used for correcting severe head–neck junction deformities, but their recommendations are still controversial [5]. AVN is the most serious complication, particularly in acute unstable SCFE, and is more frequently reported with intra-articular osteotomies for deformity correction [7]. Although the exact etiology of SCFE is unknown, several risk factors have been identified, including endocrine disorders, obesity, and excessive acetabular and/or femoral neck retroversion [8].

Treating SCFE that occurs following a femoral neck fracture presents additional challenges due to the presence of internal fixation devices and the risks associated with a nearby healing fracture site [9]. Over recent decades, only a few cases have been documented, highlighting the need for further evidence to improve treatment approaches for this complication in pediatric orthopedic trauma [10].

Virtual surgical planning (VSP), a component of Computer-Assisted Surgery (CAS), enables surgeons to simulate and plan procedures entirely within a virtual environment. It facilitates precise three-dimensional deformity analysis, implant positioning, surgical access planning, and treatment customization, enhancing intraoperative accuracy and improving postoperative outcomes [11]. When integrated with 3D printing, VSP enables the creation of patient-specific instruments (PSIs), such as anatomical models, cutting guides, and implants, which help reduce surgery time, minimize blood loss, decrease intraoperative fluoroscopy, and enhance overall efficiency compared to traditional surgery [12,13]. The increasing adoption of in-house 3D-printing facilities in hospitals further promotes personalization and lowers costs compared to industry-manufactured alternatives [14].

This case report describes the treatment of severe SCFE that occurred 1 month after surgery for a femoral neck fracture in a 15-year-old boy, and it was treated with the Imhäuser intertrochanteric osteotomy (ITO) combined with in situ fixation (ISF) and osteochondroplasty (OChP), describing how VSP with 3D-printed PSIs may be helpful for such rare and complex cases.

## 2. Case Presentation

**Figure 1:**
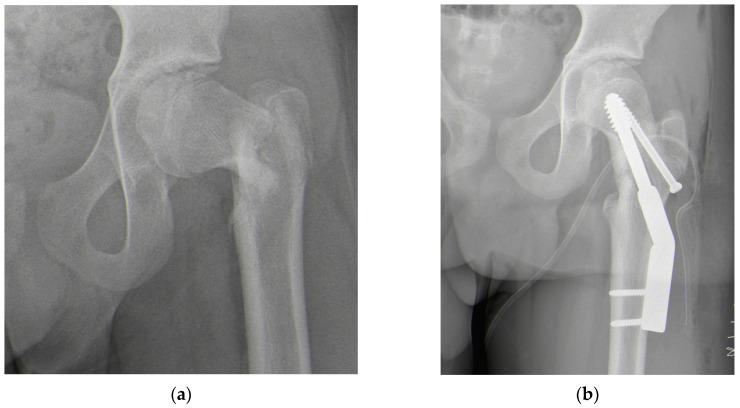

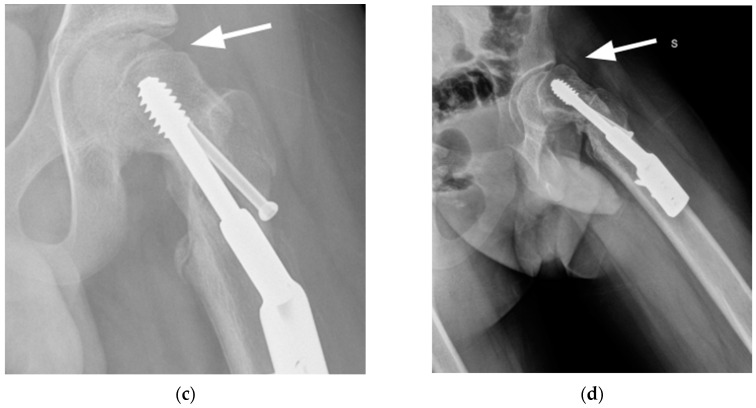
(**a**) Radiograph after trauma showing a Delbet Type III femoral neck fracture; (**b**) radiograph after open reduction and internal fixation surgery; (**c**) radiograph at one-month follow-up showing signs of mild SCFE (white arrow); (**d**) radiograph at three-month follow-up showing worsening SCFE (white arrow).

A 15-year-old male with autism spectrum disorder (ASD) associated with mild neurodevelopmental delay in a heterozygous deletion of CUX1 sustained a skiing injury in February 2024, resulting in a Delbet Type III basicervical femoral neck fracture (Figure 1a). The patient’s medical history revealed no previous fractures, surgeries, or chronic medication use. He had a BMI of 18.6 (28th percentile by age and sex) with a height of 177 cm and a weight of 55 kg. Treatment involved closed reduction and internal fixation using a Dynamic Hip Screw (DHS) plate and an additional free cannulated screw (Figure 1b). Intraoperative fluoroscopic images showed a satisfactory reduction on the anteroposterior plane with a residual 15° retroversion deformity on the lateral plane. The reduction was deemed satisfactory, and at a one-month follow-up, progressive fracture healing was noted (Figure 1c). However, three months later, radiographs revealed worsening slippage (Figure 1d). Although initially overlooked, mild slippage was evident on radiographs taken one month post-injury, with residual pain attributed to the recently healed fracture. As the condition worsened, the patient was referred to a tertiary referral center for pediatric orthopedics for further evaluation.

The patient presented with severe limitations in posture and ambulation. Radiographs revealed severe epiphysiolysis with a radiographic posterior sloping angle of 62° with the fracture healed and the fixation devices intact. The fracture had healed, and the fixation devices were in place. After a team discussion, the decision was made to remove the devices, perform ISF, and carry out an ITO with OChP of the residual bump. VSP and 3D-printed PSIs were also planned.

### 2.1. Virtual Surgical Planning and Design of 3D-Printed Cutting Guide

**Figure 2:**
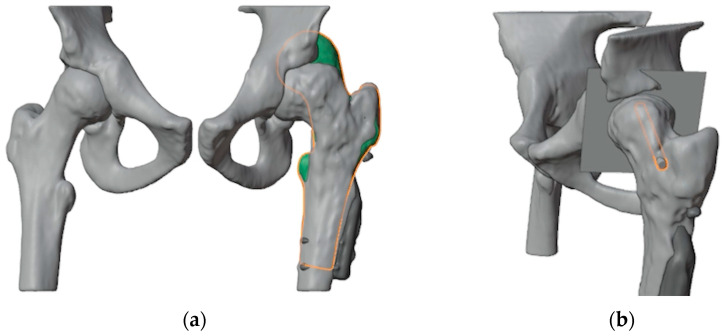
(**a**) Overlap of the healthy contralateral femur (shown in green with an orange outline); (**b**) Identification of a plane tangent to the base of the slipped epiphysis and of the position of the screw for ISF (outlined in orange).

The first step involved generating a virtual 3D model of the affected bone segments, derived from CT imaging using the hospital’s 3D-printing facility. The procedure and workflow, using Mimics Medical software Suite 25.0 (Materialise, Leuven, Belgium), have been previously described [15]. A mirrored model of the healthy contralateral hip was used to facilitate the analysis of the deformity (Figure 2a). A plane tangent to the base of the epiphysis was created in order to identify the best position of the screw for ISF. This process determined the precise entry point, direction, and length of the screw, which was 65 mm (Figure 2b). A 6.5 mm Rondò screw (Citieffe s.r.l., Calderara di Reno, Bologna, Italy) was chosen and its length was also determined.

**Figure 3:**
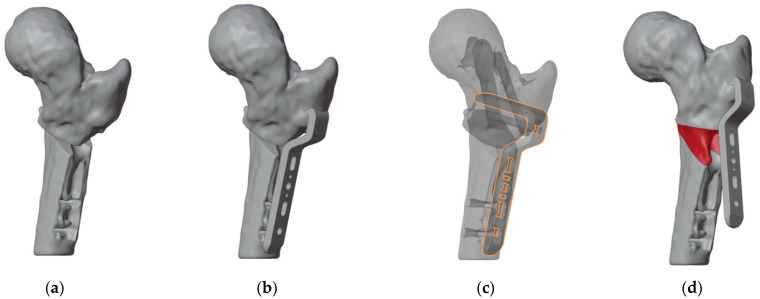
(**a**) The first step was to determine the final position of the proximal femur after an intertrochanteric closing wedge and derotative osteotomy in order to improve the range of motion of the hip; (**b**) final positioning of the 90° blade plate; (**c**) the plate (highlighted in orange) was positioned in order to avoid the holes of the previous hardware (in dark gray) as much as possible; (**d**) restoring the femur to its deformed state maintaining the plate in its position relative to the proximal femur reveals the initial position of the blade and the shape of the bone wedge that needs to be removed (in red).

To ensure precise identification of the final plate position and osteotomy sites, a reverse planning approach was employed. This began with a detailed analysis of the deformity, followed by determining the desired angular and rotational corrections to improve hip range of motion (Figure 3a). The correction was planned in valgus, flexion, and internal rotation of the distal femur. The osteotomy site and the final plate position were then meticulously defined in order to prevent intersecting the holes left by previous hardware (Figure 3b,c). We chose to use a 4.5 mm 90° Locking Cannulated Blade Plate (OrthoPaediatrics, Warsaw, IN, USA). Subsequently, maintaining the plate in its position relative to the proximal femur, the femur was reverted to its original deformed state (Figure 3d). This step was crucial for designing PSIs for positioning guidewires and for identifying the osteotomy planes.

Once cutting planes, entry points for the epiphyseal screw, and the plate position were defined, the design and fabrication of cutting guides were carried out. The guides were modeled using the CAD software Creo Parametric v7.0 (PTC Inc., Boston, MA, USA) and manufactured using Fused Deposition Modeling (FDM) 3D-printing technology.

**Figure 4:**
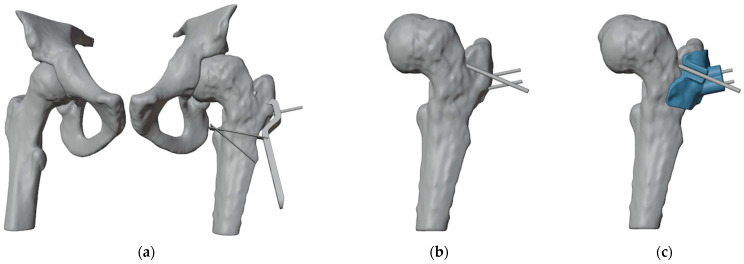
(**a**) Anterior view of the proximal femur with the initial plate positioning and the bone wedge to remove; (**b**) positioning of the guidewire for the cannulated screw (the more anterior wire) and two lateral wires for the placement of the blade plate; (**c**) design of the first 3D-printed PSI (highlighted in light blue).

The first PSI was designed to guide the insertion of three guidewires (Figure 4). The most anterior wire, with a diameter of 3 mm, was designated to position the epiphyseal screw for ISF, while the other two parallel guidewires of 1.5 mm were intended to assist in positioning the proximal portion of the plate. Notably, the design of the first guide incorporated an opening in the slot for the epiphyseal screw’s guidewire, enabling the PSI to be easily removed without interfering with the parallel wires.

**Figure 5:**
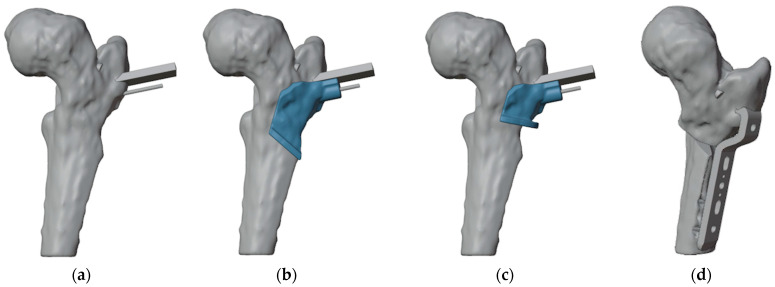
(**a**) The position of the chisel along the proximal 1.5 mm guidewire and of the distal 1.5 mm guidewire; (**b**) the second PSI(highlighted in light blue), designed to fit onto the distal guidewire, precisely indicates the directions for chisel insertion and for the distal cut; (**c**) design of the third PSI (highlighted in light blue), featuring similar characteristics to the second, but specifically guiding the proximal cut; (**d**) simulated correction in valgus, flexion, and internal rotation of the distal femur.

**Figure 6:**
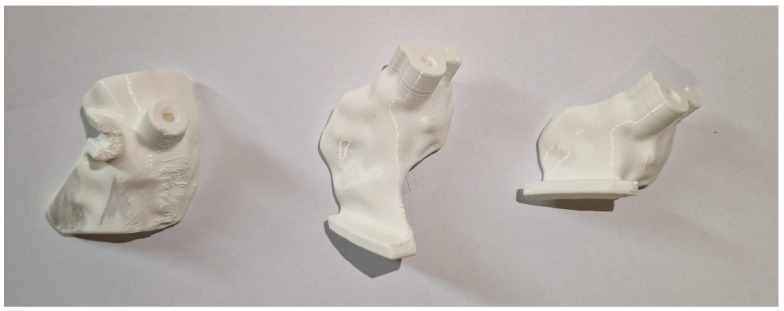
The final 3D-printed samples of the PSIs. From the left to the right: the first PSI for wire positioning, the second PSI for the distal cut, and the third PSI for the proximal cut.

Subsequently, we developed second and third PSIs to facilitate the cutting process and ensure the precise alignment of the blade plate during insertion (Figure 5). According to the surgical technique, after inserting the proximal guidewire for the blade plate, the blade slot must be prepared. To address this, both guides were equipped with a support for the chisel to achieve the desired rotation of the blade and a slot for the second guidewire, which remained unchanged. The design process started with the guide intended for the most distal cut. This guide included a slot to secure the second guidewire for accurate positioning, a recess to direct the chisel during blade slot preparation, and a raised platform to stabilize the saw for precise cutting. Next, the second guide was designed with the same features, but its saw support was specifically aligned for the more proximal cut (Figure 6).

### 2.2. Surgical Procedure

The patient was positioned on a radiolucent table with the contralateral hip flexed and abducted. The anatomical landmarks were identified, including the anterior superior iliac spine and greater trochanter, and a dotted line was drawn from the anterior superior iliac spine to the patella to assess rotational alignment on the skin. Prior to the procedure, anteroposterior, lateral, and oblique views in internal and external rotation were verified using an image intensifier.

**Figure 7:**
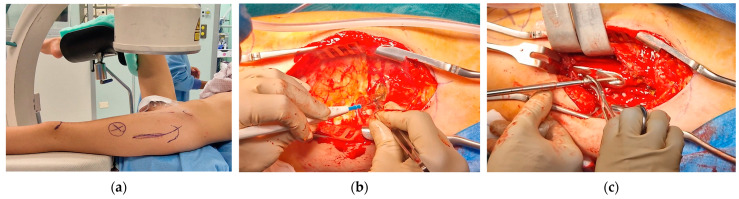
(**a**) Anatomical landmarks and fluoroscopy check; (**b**) L-incision along the proximal inferior border of the vastus lateralis; (**c**) removal of the DHS plate and of the proximal anti-rotation screw.

A curvilinear lateral incision that incorporated the previous scar was marked. The previous scar was excised, and the subcutaneous tissue was retracted to expose and open the fascia lata in line with the skin incision. An L-shaped incision was made along the proximal inferior border of the vastus lateralis, facilitating subperiosteal exposure of the proximal femur. The DHS plate was subsequently identified and removed, along with the free screw (Figure 7).

**Figure 8:**
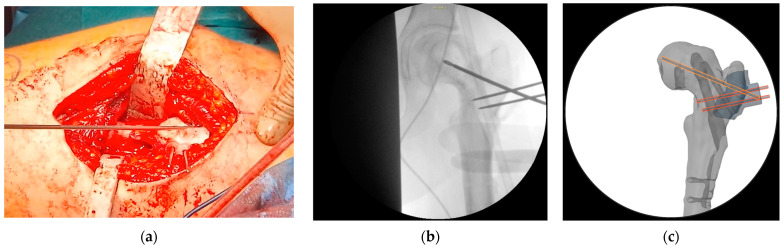
The intraoperative application of the first PSI. (**a**) Intraoperative picture of the first PSI in place; (**b**) intraoperative imaging of guidewire positioning; (**c**) position of guidewires for the free screw for ISF (highlighted in yellow) and for the blade plate (highlighted in orange) in the VSP for comparison with the intraoperative imaging.

At this point, the hip was slightly externally rotated to facilitate the positioning of the first patient-specific template (Figure 8). The three guidewires were inserted, the template removed, and the 6.5 mm cannulated epiphyseal screw easily placed along the 3 mm guidewire. The 3 mm guidewire was then removed.

**Figure 9:**
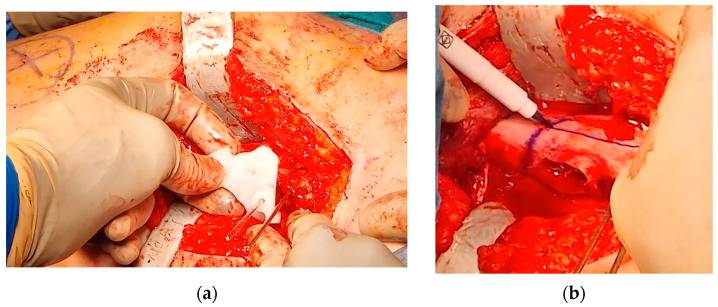
The intraoperative application of the second PSI. (**a**) The distal 1.5 mm guidewire was leveraged to precisely fit the second PSI; (**b**) a longitudinal line was marked to monitor rotational alignment.

The second PSI was positioned on the femur, leveraging the distal 1.5 mm guidewire as a reference to accurately identify the distal osteotomy site (Figure 9). A longitudinal line was marked along the anterior part of the diaphysis to assess rotational alignment.

**Figure 10:**
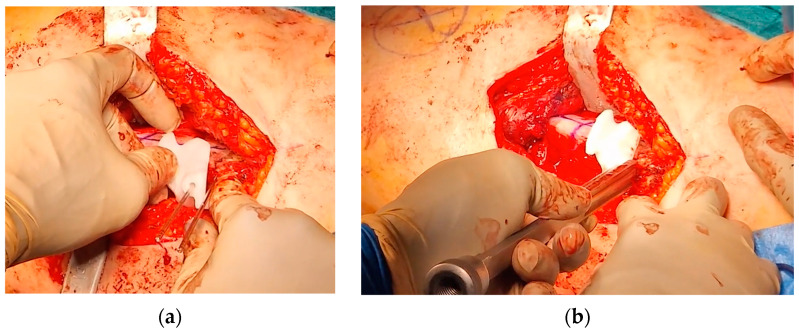
The intraoperative application of the third PSI. (**a**) Application of the third cutting guide on the previously inserted guidewire; (**b**) application of the third guide to set the correct angulation of the chisel.

The third PSI was then applied to identify the proximal osteotomy and to achieve the correct angulation of the chisel in the sagittal plane, allowing for the placement of the blade plate at 20° of flexion (Figure 10). The cannulated chisel was inserted along the proximal guidewire, while the PSI was still secured to the femur using the distal 1.5 mm guidewire. Notably, both the second and third PSIs could be utilized to guide the chisel, providing a reliable backup in case of device issues such as breakage or contamination.

Once the chisel was in its final position, the third PSI was carefully removed, and the second PSI was reinserted to verify the positions of the two cuts. The distal cut was then marked using an oscillating saw, but not completed. This step facilitated the internal rotation of the distal fragment along the proximal straight osteotomy.

The third PSI was reapplied, and the proximal femoral cut was completed. Afterward, the femur was internally rotated, the chisel removed, and the blade plate gently inserted, securing it with the proximal locking screw to prevent displacement and cut-out.

Next, the distal cut was completed with the femur internally rotated, and an anterolateral bony wedge was removed. The thigh was flexed and abducted, securing the distal part of the blade plate to the femoral shaft with a clamp. The correct position and orientation of the osteotomy were verified, and the plate was fixed with screws.

**Figure 11:**
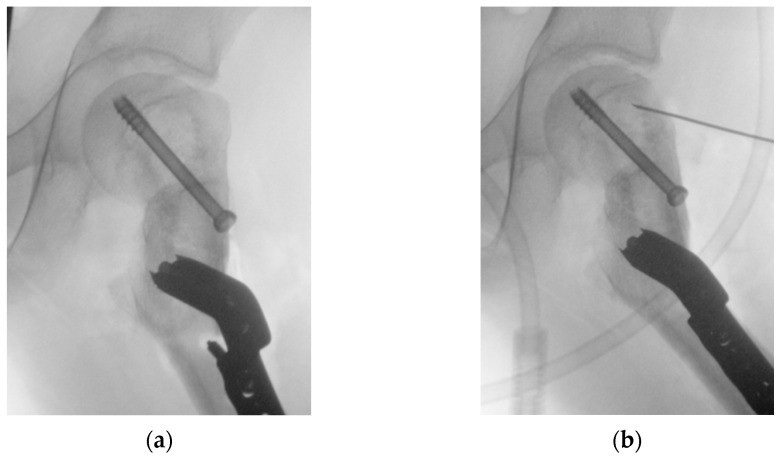
(**a**) Intraoperative fluoroscopy showing the anterior bump; (**b**) intraoperative fluoroscopy showing the bump removal after the OChP (fine needle marks the area of the resected bump).

After completing the osteotomy, the proximal femur was shifted anteriorly and laterally, improving visibility and access to the residual bump. Taking advantage of the Watson–Jones anterolateral approach, the anterior edges of the gluteus medius and gluteus minimus were partially detached, and the anterior fat pad was elevated to expose the joint capsule. An anterolateral reverse T-shaped capsulotomy was performed. The residual anterolateral bump was identified through visual inspection and fluoroscopic guidance, and was carefully removed using straight and curved osteotomes, rongeurs, and burs. A final fluoroscopic check confirmed the satisfactory removal of the intra-articular bump (Figure 11).

The joint capsule was then closed, the muscles were reattached, and the skin was sutured.

### 2.3. Postoperative Protocol

The postoperative rehabilitation protocol included passive and active hip motion in flexion and abduction, avoiding hip rotations for the first six weeks. Sitting, standing, and early ambulation with partial weight-bearing were allowed from the day after surgery and continued for six weeks. No cast or brace was required. From 6 to 12 weeks, full weight-bearing was gradually achieved, and full active range of motion of the hip was restored; swimming and biking were recommended. From 12 to 24 weeks, muscle-strengthening exercises, gait improvement, and progressive return to running and jumping were introduced. Return to sports was allowed six months after surgery.

### 2.4. Results

**Figure 12:**
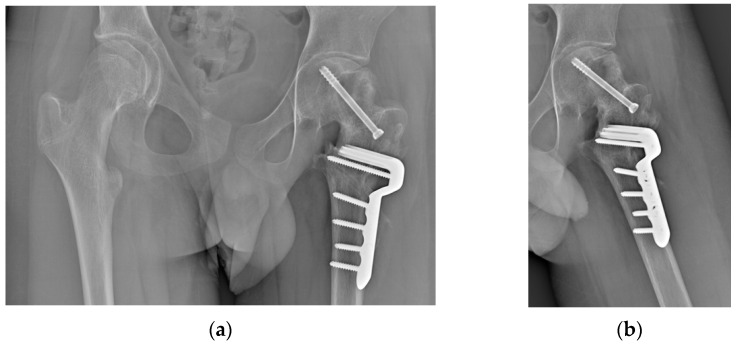
Radiographs at 6 months follow-up. (**a**) Anteroposterior view; (**b**) frog-leg view.

The presented case required approximately two weeks from the date of the preoperative CT scan for the planning, design, production, and sterilization of the PSIs. The surgery was performed without any complications. Blood loss was 500 mL, the number of fluoroscopic images was 41 (cumulative dose 72.7 cGy/cm^2^), and the surgical time was 176 min. At the last follow-up, 6 months postoperatively, the patient had resumed walking, although with difficulties in following the rehabilitation program due to his autism. The hip was highly mobile and pain-free. Radiographs showed good consolidation with no signs of AVN of the femoral head (Figure 12).

## 3. Discussion

This case report described a SCFE following a femoral neck fracture in an adolescent. Both events are rare in this age group, and their combined occurrence has been documented in fewer than ten cases in the literature (see Table 1 for details) [9,10,16,17,18,19,20,21,22]. SCFE may develop up to 15 months post-trauma, and it has been frequently reported in children below 10 years of age [9,10,17,18,21]. Based on Loder and Skopelja’s observation that the average age for SCFE onset is 12.0 years in males and 11.2 years in females, with a trend toward younger ages, we might hypothesize that SCFE could be a complication associated with femoral neck fractures in younger patients [23]. However, the case presented here notably increases the age range at which this complication may arise. In our case, we believe that a slight initial malreduction, leaving the neck in mild retroversion, may have increased stress on an already fragile physis. We do not think excess weight played a role, as the patient was very thin (BMI 18.6, 28th percentile by age and sex), although SCFE in non-obese adolescents has been reported to occur at older ages [24]. Furthermore, the CUX1 gene mutation, identified as the underlying cause of the patient’s neurodevelopmental disorder, has not previously been linked to any bone or cartilage disorders. Another consideration is the choice of fixation, which avoided crossing the physis with cervical screws. While this is the standard choice for children to prevent femoral neck shortening, many believe that crossing the physis in older children poses minimal risk of shortening and it can improve fixation stability, preventing such complications [25].

In nearly all other cases of SCFE following femoral neck fracture reported in the literature, epiphyseal fixation was performed, occasionally accompanied by closed reduction maneuvers. Additionally, an intertrochanteric valgus osteotomy was carried out in four cases (44%). No cases of open reduction and/or modified Dunn procedures were reported. There is ongoing debate on the best strategy to restore joint alignment in moderate to severe slips [5]. Many authors believe intra-articular osteotomies are more effective because they correct the deformity at its source [26]. However, these procedures are technically challenging and carry a significant risk of AVN [7,27]. In contrast, extra-articular osteotomies are simpler to perform, and have a lower risk of complications such as AVN, as they do not compromise the terminal blood supply to the femoral epiphysis [7,27]. However, they are less effective in correcting femoral deformity, potentially leaving residual bumps that can reduce hip mobility, cause femoro-acetabular impingement, and lead to early osteoarthritis [27]. In our experience, we have confirmed lower rates of AVN and early complications with extra-articular osteotomies but also less effective deformity correction compared to the modified Dunn procedure [28]. We believe extra-articular osteotomy is preferable in cases like this, where the posterior periosteum of the femoral neck, containing the terminal vessels of the epiphyseal blood supply, has already been partially damaged by the fracture and its fixation. Exposing it to further stress from a modified Dunn procedure could have increased the risk of AVN.

We have previously detailed our extensive experience with the Imhäuser ITO [29]. This procedure corrects the position of the femoral epiphysis and restores joint alignment by adjusting the proximal femur in three planes: valgus, flexion, and external rotation. Our earlier studies have shown satisfactory results, with native hip survival at 20, 30, and nearly 40 years of follow-up, and a low risk of necrosis, chondrolysis, and early total hip replacement. These satisfactory results are consistently supported by numerous studies in the literature [7,30,31,32,33,34,35,36,37,38].

The use of virtual surgical planning and PSIs for osteotomies in pediatric orthopedic surgery has demonstrated a significant reduction in surgery time and decreased reliance on intraoperative imaging [39,40,41,42]. The reduced use of fluoroscopic imaging serves as an indirect indicator of the efficiency and precision with which correction and fixation are achieved. This support is especially valuable during the Imhäuser intertrochanteric osteotomy, as both the correction and fixation positioning are counterintuitive [43,44]. Having this technology in-house, using low-cost methods and standard implants, lowers costs while maintaining high surgical safety and precision [42,45].

Personalized treatments and patient-specific instruments (PSIs) offer significant benefits but come with higher costs due to the need for advanced materials, specialized technologies, and skilled personnel. In-hospital 3D-printing facilities can help reduce these expenses [46,47]. Our previous experience suggests that in-hospital VSP and 3D printing improve cost–utility, especially in complex surgeries, by reducing surgical time, complications, and implant errors, while enhancing outcomes and minimizing reoperations. However, the process still requires substantial resources, including expertise, software, printers, and efforts in design, production, and sterilization. We are continuing research to reduce the economic impact of this promising technology, including integrating immersive virtual reality, augmented reality, and AI-enhanced planning [48].

It has been clearly demonstrated that assessing cam deformity in SCFE surgery is essential to prevent acetabular damage and ensure long-lasting hip function [49]. Some authors have even proposed combining the Imhäuser intertrochanteric osteotomy with a safe hip dislocation to enhance visualization and remodeling of the femoral head [38,50]. However, for the recent fracture, we opted to avoid dislocating the head and instead perform OChP through an anterolateral capsulotomy, according to the technique reported by Abdelaziz et al. [51]. We noted that there was no mention of additional treatments for the anterior bump in other cases of SCFE following a femoral neck fracture.

Despite being a case report, this study adds valuable evidence on a complication of femoral neck fractures, which is probably underappreciated and underreported [22]. This case highlights a rare complication of pediatric femoral neck fractures, stressing the importance of perfect fracture reduction and the potential benefit of combined ISF of the femoral epiphysis. In the debated treatment of severe SCFE, we advocate for ITO with ISF as the preferred approach over techniques like the modified Dunn procedure, especially when associated with a recent femoral neck fracture.

## 4. Conclusions

The Imhäuser intertrochanteric osteotomy, combined with in situ fixation and osteochondroplasty, proved to be a reliable surgical option for severe SCFE following a femoral neck fracture. Among the cases reported in the literature, there is still no evidence of major complications, such as avascular necrosis and/or early conversion to total hip replacement. The use of virtual surgical planning and 3D-printed surgical guides in the Imhäuser intertrochanteric osteotomy can significantly enhance the accuracy of correction compared to the classic approach. Additionally, the residual intra-articular bump can be safely and effectively removed concurrently through an anterolateral approach.

## Figures and Tables

**Table 1:** Cases of slipped capital femoral epiphysis (SCFE) after femoral neck fractures described in the literature by year of publication. M = male; F = female; CR = closed reduction; OR = open reduction; ISF = in situ fixation; VO = valgus osteotomy; VFO = valgus and flexion osteotomy; OChP = osteochondroplasty.

Author and Year	Age (Years)	Sex	Delbet Type	Treatment for Fracture	Fracture to SCFE (Months)	SCFE Severity	Treatment for SCFE
Ogden et al., 1975 [16]	11	M	II	CR + cast	15	Mild	None
Manukaran et al., 1989 [17]	9	M	III	CR + screws	14	Mild	ISF
Joseph and Mulpuri, 2000 [18]	3.8	M	II	CR + screws	1	Moderate	CR + pins + VO
Gopinathan et al., 2012 [19]	10	M	II	CR + screws + cast	4	Mild	CR + screw + cast
Jung and Park, 2012 [20]	11	M	III	OR + screws + splint	15	Mild	ISF + VFO + cast
Li et al., 2013 [9]	12	F	III	CR + screws + cast	5	Moderate	CR + pins
Li et al., 2013 [9]	6	F	II	CR + plate	9	Mild	ISF + VO
Chinoy et al., 2020 [10]	5	F	III	CR + cast	7	Moderate	ISF
Elbaseet et al., 2023 [21]	9	F	III	OR + screws	3	Moderate	ISF + VO
Current study, 2024	15	M	III	CR + plate	1	Severe	ISF + VFO + OChP

## Data Availability

The data presented in this study are available on request from the corresponding author due to the patient’s privacy.
